# Coherence of BOLD signal and electrical activity in the human brain during deep sevoflurane anesthesia

**DOI:** 10.1002/brb3.679

**Published:** 2017-05-17

**Authors:** Daniel Golkowski, Andreas Ranft, Tobias Kiel, Valentin Riedl, Philipp Kohl, Guido Rohrer, Joachim Pientka, Sebastian Berger, Christine Preibisch, Claus Zimmer, George A. Mashour, Gerhard Schneider, Eberhard F. Kochs, Rüdiger Ilg, Denis Jordan

**Affiliations:** ^1^ Department of Neurology Klinikum rechts der Isar der Technischen Universität München München Germany; ^2^ Department of Anesthesiology Klinikum rechts der Isar der Technischen Universität München München Germany; ^3^ Department of Neuroradiology Klinikum rechts der Isar der Technischen Universität München München Germany; ^4^ Department of Anesthesiology University of Michigan Medical School Ann Arbor MI USA; ^5^ Department of Neurology Asklepios Kliniken Bad Tölz Germany

**Keywords:** BOLD, burst suppression, EEG, neurovascular coupling, sevoflurane

## Abstract

**Introduction:**

Changes in neural activity induce changes in functional magnetic resonance (fMRI) blood oxygenation level dependent (BOLD) signal. Commonly, increases in BOLD signal are ascribed to cellular excitation.

**Objective:**

The relationship between electrical activity and BOLD signal in the human brain was probed on the basis of burst suppression EEG. This condition includes two distinct states of high and low electrical activity.

**Methods:**

Resting‐state simultaneous EEG and BOLD measurements were acquired during deep sevoflurane anesthesia with burst suppression EEG in nineteen healthy volunteers. Afterwards, fMRI volumes were assigned to one of the two states (burst or suppression) as defined by the EEG.

**Results:**

In the frontal, parietal and temporal lobes as well as in the basal ganglia, BOLD signal increased after burst onset in the EEG and decreased after onset of EEG suppression. In contrast, BOLD signal in the occipital lobe was anticorrelated to electrical activity. This finding was obtained consistently in a general linear model and in raw data.

**Conclusions:**

In human brains exhibiting burst suppression EEG induced by sevoflurane, the positive correlation between BOLD signal and electrical brain activity could be confirmed in most gray matter. The exceptional behavior of the occipital lobe with an anticorrelation of BOLD signal and electrical activity might be due to specific neurovascular coupling mechanisms that are pronounced in the deeply anesthetized brain.

## 1 INTRODUCTION

Functional magnetic resonance imaging (fMRI) is probably the most widely used technique in functional imaging of the human brain and uncovers local hemodynamic changes that are thought to mirror electrical brain activity. A considerable amount of research has been carried out to shed light on the physiological principles that underlie BOLD signal changes. In animal models using visual stimulation, BOLD signals were found to reflect local field potentials (e. g., Logothetis, Pauls, Augath, Trinath, & Oeltermann, 2001; Rauch, Rainer, & Logothetis, 2008; Viswanathan & Freeman, 2007). Relating BOLD signals to EEG frequency bands yielded a positive correlation with gamma band power following visual stimulation (Goense & Logothetis, 2008; Kayser, Kim, Ugurbil, Kim, & Konig, 2004; Niessing et al., 2005) and also during spontaneous activity (Magri, Schridde, Murayama, Panzeri, & Logothetis, 2012; Murayama et al., 2010; Scholvinck, Maier, Ye, Duyn, & Leopold, 2010). This connection was validated in humans performing a cognitive task during simultaneous EEG‐fMRI recording where BOLD signal fluctuations were positively correlated with high gamma (60–80 Hz) power and negatively correlated with alpha and beta power (Scheeringa et al., 2011). Such a negative correlation of BOLD signal changes and EEG band power below 30 Hz could also be observed during the resting state (Laufs et al., 2006) as well as during task paradigms (Scheeringa et al., 2009; Yuan et al., 2010) in combined EEG‐BOLD measurements. A strictly data‐driven approach to simultaneous EEG‐fMRI measurements in human brains revealed distinct electrophysiological signatures of resting state networks, corroborating that the various frequency bands differentially influence BOLD signal (Mantini, Perrucci, Del Gratta, Romani, & Corbetta, 2007).

In order to further characterize the BOLD response, we chose a condition that includes an extensive interruption of ongoing activity. Burst suppression (BS), an EEG pattern that occurs during deep anesthesia as well as in certain pathological conditions, is characterized by randomly alternating phases of generalized high amplitude activity and phases of quiescence (Brown, Lydic, & Schiff, 2010; Japaridze et al., 2015). Sevoflurane, a halogenated ether widely used for surgical anesthesia, is able to generate such phases of burst and suppression, each lasting for several seconds to minutes. The contrast between the two states enables the investigation of the relationship of BOLD signal and electrical activity. Unlike the so‐called resting state, which is characterized by task‐independent ongoing neuronal activity, BS uniquely offers the opportunity to oppose two different conditions with particular neuronal activity while head motion is minimized.

We used sevoflurane‐induced BS in human volunteers to investigate the relation between EEG activity and BOLD signal.

## 2 MATERIAL AND METHODS

The presented data were obtained from a study on sevoflurane‐induced unconsciousness (Ranft et al., 2016). In the following paragraphs, acquisition of the data for the present study of BS is briefly described.

### 2.1 Study participants

The ethics committee of the medical department of the Technische Universität München (München, Germany) approved the present study, and the study protocol was in accordance with the Declaration of Helsinki. Volunteers were given detailed information about the protocol and risks, and written informed consent was obtained at least 48 hr before the experimental session; volunteers were reimbursed for their participation. Twenty healthy adult males aged 20–36 (mean, 26) years were recruited by means of notices posted on campus and via personal contact. Prior to inclusion in the study, a medical history was taken to identify any contraindication against the planned procedure (physical status other than American Society of Anesthesiologists physical status class I, chronic intake of medication or drugs, hardness of hearing or deafness, absence of fluency in German, known or suspected disposition to malignant hyperthermia, acute hepatic porphyria, a history of halothane hepatitis, obesity with a body mass index >30 kg/m^2^, gastrointestinal disorders with a disposition for gastroesophageal regurgitation, known or suspected difficult airway, presence of metal implants). A focused physical examination was performed and a resting electrocardiogram was recorded. Experiments were conducted between June and December 2013.

### 2.2 Study protocol

Participants were supine on the scanner table. After inserting an intravenous catheter in a vein on the dorsum of the hand, sevoflurane in oxygen was administered via a tight fitting facemask using an fMRI‐compatible anesthesia machine (Fabius Tiro, Dräger, Lübeck, Germany). Sevoflurane as well as O_2_ and CO_2_ were measured by the cardiorespiratory monitor (Datex AS/3; General Electric, Fairfield, CT, USA); standard American Society of Anesthesiologists monitoring was performed. An end‐tidal sevoflurane concentration (etSev) of 0.4 vol% was administered for 5 min, then increased in a stepwise fashion until the participant was unconscious. Sevoflurane concentration was then increased to reach an end‐tidal concentration of approximately 3 vol%.

When clinically indicated, ventilation was managed by the physician and a laryngeal mask suitable for fMRI (i‐gel, Intersurgical, Wokingham, UK) was inserted. FiO_2_ was then set at 0.8 and mechanical ventilation was adjusted to maintain end‐tidal CO_2_ at a steady concentration of 33 ± 1.71 mmHg and SpO_2_ at 98 ± 0.66% (in this paragraph, mean ± standard deviation). Norepinephrine was given by continuous infusion (0.1 ± 0.01 μg/kg*min) to maintain the mean arterial blood pressure close to baseline values (baseline 96 ± 9.36 mmHg, BS 88 ± 7.55 mmHg). After insertion of the laryngeal mask, sevoflurane concentration was gradually increased until the EEG showed BS with suppression periods of at least 1000 ms (reached at 4.34 ± 0.22 vol%). At that point, a data set containing 700 s of simultaneous EEG and fMRI was recorded.

In order to monitor the subject's recovery from anesthesia, the patient table was slid out of the scanner gantry and sevoflurane administration terminated. The volunteer was at that point manually ventilated until spontaneous ventilation returned. The laryngeal mask was removed as soon as the patient opened his mouth on command. When participants were alert, oriented, cooperative, and had stable vital signs, they were taken home by a family member or a friend appointed in advance.

### 2.3 EEG data acquisition and analysis

Simultaneous EEG‐fMRI recordings were performed using an fMRI‐compatible, 64‐electrode cap with equidistantly arranged ring‐type sintered nonmagnetic Ag/AgCl electrodes (Easycap, Herrsching, Germany) and two 32‐channel, nonmagnetic, battery‐operated EEG amplifiers (BrainAmp MR; Brain Products, Gilching, Germany). Electrode impedance was kept below 5 kΩ using an abrasive gel (Easycap). An interface unit (SyncBox; Brain Products) was additionally connected to the amplifiers to reduce timing‐related errors in the fMRI artifact correction by synchronizing the clocks of the EEG amplifiers and the fMRI gradients. One of the 64 channels was placed over the chest and registered the electrocardiogram (left anterior axillary line). All signals were recorded at 5 kHz sampling rate (BrainVision Recorder; Brain Products). The EEG signal preprocessing analyses were performed using BrainVision Analyzer 2 (Brain Products). fMRI gradient artifacts in the EEG were averaged using a sliding window and subtracted from the EEG signals (Allen et al., 2011). The cardioballistic artifacts caused by cardiomechanic electrode induction were removed using a template‐detection method. The templates were based on the detected local maxima (R‐peaks) and subtracted from the EEG using sliding windows of 21 epochs (Allen et al., 2011).

Each fMRI volume was assigned to either burst or suppression according to the simultaneously recorded EEG traces, which were analyzed visually by a trained student and categorized in either burst or suppression. An almost flat line EEG with low power in all frequency bands characterized suppression phases. Burst phases were always initiated by generalized typical burst activity (cf. Brown et al., 2010) and then changed into high amplitude activity mainly from the delta band. However, for the purposes of this study, we designated the whole non‐suppression phase as burst. Volumes were regarded as transition between both states if the corresponding EEG was not attributable to a single state for the entire 2 s repetition time.

### 2.4 fMRI acquisition and analysis

Data acquisition was carried out on a 3‐Tesla whole‐body MRI scanner (Achieva Quasar Dual 3.0T 16CH, Amsterdam, the Netherlands) with an eight‐channel, phased‐array head coil. The data were collected using a gradient echo planar imaging sequence (echo time = 30 ms, repetition time = 2000 ms, flip angle = 75°, field of view = 220 × 220 mm, matrix = 72 × 72, 32 slices, slice thickness = 3 mm, and 1 mm interslice gap; 700 s acquisition time, resulting in 350 volumes with 3 × 3 × 3 mm voxel size). Anatomy was acquired before the functional scan using a T1‐weighted sequence and 1 × 1 × 1 mm voxel size.

Data were preprocessed using Statistical Parametric Mapping (SPM8, Wellcome Trust Centre for Neuroimaging, University of London, UK) and Data Processing Assistant for Resting‐State fMRI (Chao‐Gan & Yu‐Feng, 2010). Functional and T1‐weighted images were first reoriented manually and realigned by means of SPM8 to the mean image of all functional images. Next, the first three volumes were removed and slice timing was corrected. Functional images and structural images were coregistered to the standard fMRI template implemented in SPM8 and resliced to 2 × 2 × 2 mm voxel size using a 3rd degree spline interpolation. Afterwards they were normalized to Montreal Neurological Institute (MNI) space. Functional images were smoothed using a 3 × 3 × 3 mm Gaussian kernel. Since movement between volumes during BS did not exceed 2 mm in translation or 0.02° in rotation, we considered it appropriate not to exclude any data.

The general linear model (GLM) in single subjects was modeled in SPM8 with appearance of burst (binary with 1 for burst and 0 for suppression or transition) and convoluted with the canonical hemodynamic response function (HRF) implemented in SPM8 as regressor of interest and six movement parameters (linear motion and rotation with respect to x‐, y‐ and z‐axes) as nuisance regressors. We opted for this conservative and widely used approach because the same analysis without movement regressors resulted in larger negatively correlated areas. For group analysis, we applied second level statistics implemented in SPM (random effects model) using the one‐sample t‐test without cluster threshold. Results were deemed significant for uncorrected *p *< .001 and plotted over an anatomic example from the cohort using MRICron (Chris Rorden Laboratory, University of South Carolina, USA).

For the region of interest (ROI)‐based analysis, the mean time course of the voxels belonging to a given ROI (from the co‐registered Harvard‐Oxford atlas) was extracted. ROIs were chosen based on the activation maps generated by the GLM since we did not have an a priori hypothesis concerning negative correlations between burst appearance on the EEG and BOLD signal decrease.

## 3 RESULTS

### 3.1 Burst suppression EEG

**Figure 1:**
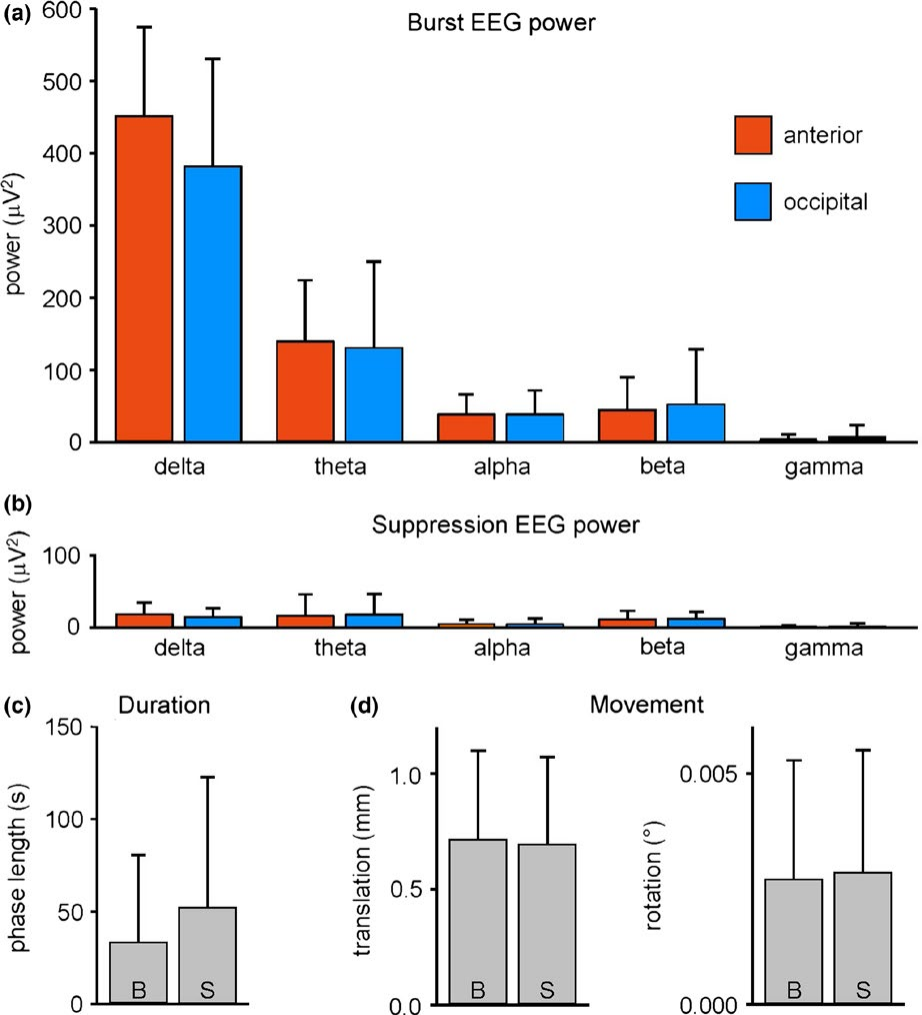
(a) Burst and (b) suppression EEG signal power of the delta [0.5 Hz, 4 Hz], theta [4 Hz, 8 Hz], alpha [8 Hz, 12 Hz], beta [12 Hz, 30 Hz], and gamma band [30 Hz, 70 Hz] in pooled anterior (frontal, parietal, and temporal) and pooled occipital electrodes (frequency analyses of EEG signals with a minimum of 30 s length were included into the plots, resulting in data from 11 subjects). (c) Length of all 145 burst and all 147 suppression phases included in fMRI group analysis (resulting in data from 19 subjects). (d) Head movement shown as maximum translation and rotation occurring in the burst and suppression phases. All bars are means + *SD*; B burst, S suppression

The EEG pattern of BS was reached in all participants. Visual classification of the EEG signals in burst and suppression phases resulted in a different signal power for both phases, that is, enhanced power in all frequency bands during burst phases (Mann–Whitney *U* test at threshold *p *< .05, Figure 1a) when compared to power in suppression phases (Figure 1b). However, the power spectrum did not differ significantly between anterior and occipital electrodes (Mann–Whitney *U* test at threshold *p *< .05, Figure 1a,b). According to the EEG, each volume of the simultaneously acquired fMRI was assigned to either burst or suppression, and fMRI data were analyzed as a function of these two EEG states. fMRI volumes were attributed to 55.1% suppression, 39.5% burst, and 4.3% transition (1.1% not classifiable). Average length of burst and suppression phases was 33 ± 4.0 s and 52 ± 5.8 s, respectively (Figure 1c). One data set could not be used because, during recording, EEG remained in suppression and no further bursts occurred, resulting in *n *= 19 subjects included in fMRI analysis. Head motion was small and did not differ significantly between burst and suppression phases (Mann–Whitney *U* test at threshold *p *< .05, Figure 1d).

### 3.2 General linear model analysis

**Figure 2:**
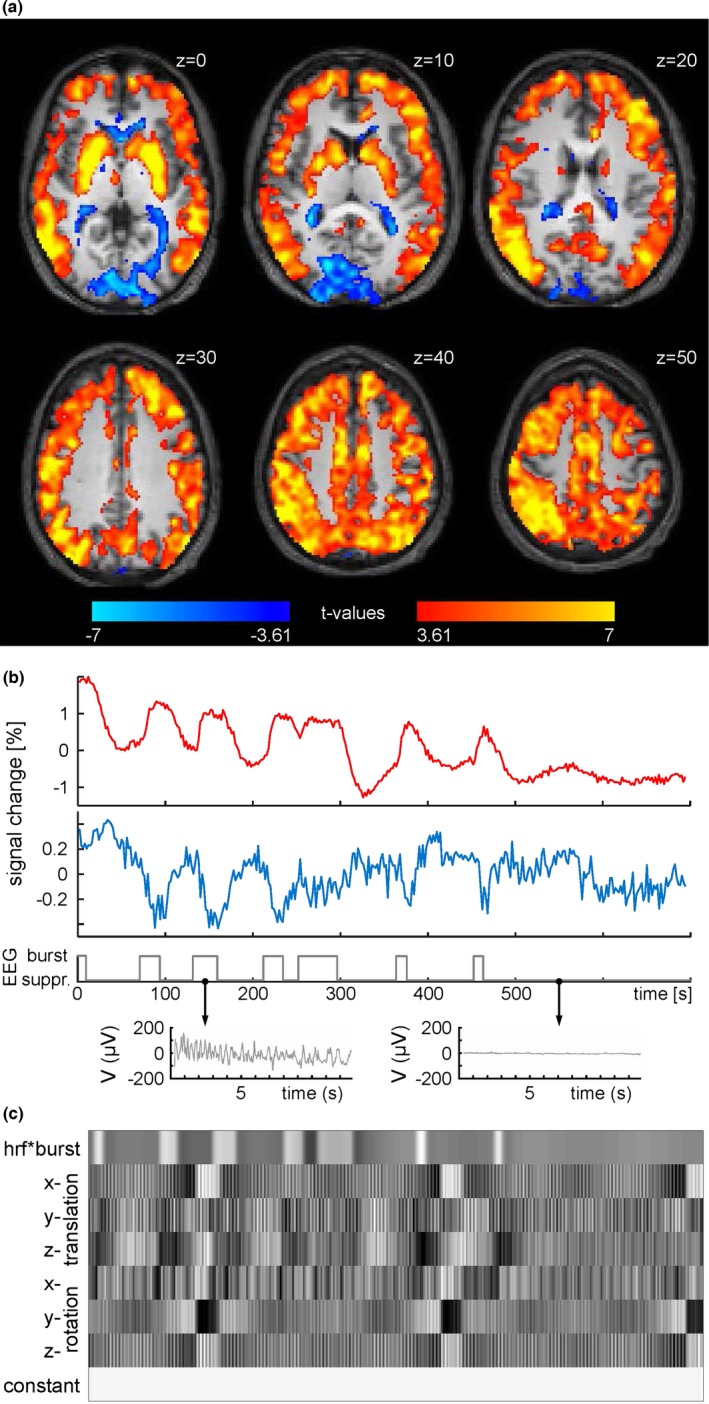
(a) Group statistics (*n *= 19) shown as voxel‐wise t‐values of a GLM modeling burst on the EEG as regressor of interest in a 700 s dataset in warm colors and an opposing contrast (“−1”) modeling anticorrelated signal changes in cold colors (347 volumes, one‐sample t‐test, *p *< .001, uncorrected). *t *= 3.61 corresponds to *p *= .001; *t *= 7 corresponds to *p *= .000008. Axial brain slices at z positions (according to MNI system) as indicated. (b) Raw time courses of ROIs from Harvard‐Oxford Atlas. Red trace: bilateral caudate nucleus. Blue trace: bilateral intracalcarine cortex. Gray trace: EEG classification and detail view of the recording from an occipital electrode. (c) Matrix for the subject also shown in (b), illustrating the individual GLM which includes burst appearance convoluted with the canonical HRF, six movement parameters and a constant term

For a statistical analysis on group level (*n *= 19), we modeled a HRF on the appearance of burst in the EEG. Although movements were below 2 mm (translation) and 0.02° (rotation) in all subjects during burst as well as suppression phases, all six movement parameters were included as nuisance regressors. Group analysis revealed that BOLD activity in most of the gray matter (cluster size, 81,600 voxels) significantly increased in association with burst phases on the EEG (Figure 2a, height threshold *p *< .001 uncorrected, warm colors). Greatest *t*‐values were observed in the basal ganglia (*t *= 13.05 at MNI coordinate 24 −2 2 mm). By contrast, BOLD signal in the occipital cortex did not demonstrate a significant coactivation with respect to the electrical signal. Inversion of the contrast revealed that the BOLD signal of the occipital cortex was time locked to the appearance of suppression on the EEG (Figure 2a, height threshold *p *< .001 uncorrected, cold colors). Anatomically, this negatively correlated cluster (size, 3909 voxels) projected onto the cuneus with a maximum around the calcarine sulcus (*t *= 7.92 at MNI coordinates −19 −96 4 mm). None of the subjects showed a positively correlated BOLD signal in the occipital cortex, while a negatively correlated signal could be seen in 14 out of 19 subjects. Thalamic activity could not be associated with either of the two interaction patterns, in contrast to the notion of its strong involvement in burst generation (Brown et al., 2010; Liu, Zhu, Zhang, & Chen, 2011).

In order to illustrate raw data as averaged time courses, the bilateral caudate nucleus and the bilateral intracalcarine cortex were chosen as ROIs from the Harvard‐Oxford atlas. These two regions demonstrated opposite relationships of BOLD signal and EEG activity, which were maintained over the whole recording period on a single subject level (Figure 2b). Since burst and suppression phases are unpredictable with respect to time of appearance and length, an individual GLM had to be constructed for each subject (an exemplary matrix is shown in Figure 2c).

### 3.3 Kinetics of BOLD response

**Figure 3:**
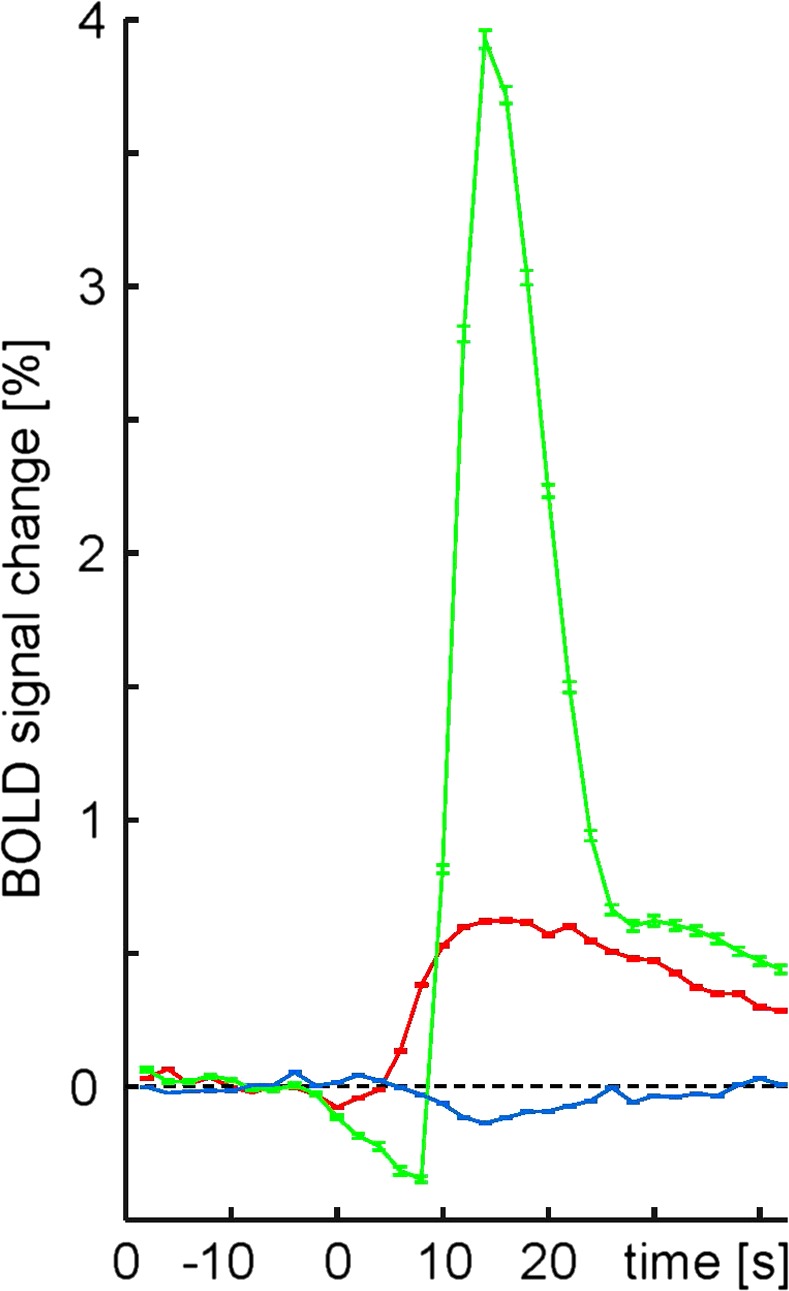
Averaged time courses of BOLD signal amplitudes (mean ± *SD*) with respect to burst onset (at *t *= 0). Green, model hemodynamic response from SPM; red, bilateral caudate nucleus; blue, bilateral intracalcarine sulcus of Harvard‐Oxford Atlas. 139 burst phases were included. Each recording was normalized to mean intensity before burst onset

The kinetics of the average positive and negative BOLD signal is shown in Figure 3. Average peak amplitude across all gray matter voxels positively correlated with burst appearance was 1.14% and peak of negatively correlated gray matter voxels was −0.48%. Mean time to peak was 26 s for positive BOLD changes and 22 s for negative signal changes. The model hemodynamic response from SPM had a much larger amplitude of 4.44% and time to peak of 13 s. This is consistent with the observation that sevoflurane reduces BOLD signal amplitude (Palanca et al., 2015).

## 4 DISCUSSION

In the present study, brains of human subjects profoundly anesthetized with sevoflurane exhibited a close coupling of EEG and BOLD signal across the cerebral cortex and subcortical structures. In the frontal, parietal, and temporal lobe as well as basal ganglia, the two signals were positively correlated, while there was an anticorrelation in the occipital lobe. This was found by means of a GLM and confirmed by individual time series analyses.

fMRI BOLD depends on the interplay between cerebral blood flow (CBF), cerebral blood volume (CBV), and the cerebral metabolic rate of oxygen (CMRO_2_) (Kim & Ogawa, 2012; Ogawa, Lee, Kay, & Tank, 1990), which are all related to neuronal activity. These variables are influenced by volatile anesthetics like isoflurane and sevoflurane (Kaisti et al., 2003). At sedative concentrations of sevoflurane (0.5 vol%), CBF and BOLD signal were positively correlated, but the slope of this relation depended on the brain region (Qiu, Ramani, Swetye, & Constable, 2008). In subjects rendered unresponsive by sevoflurane (1.2 vol%), the amplitude of BOLD signal fluctuations was globally reduced (Palanca et al., 2015). Still, neurovascular coupling seems to be qualitatively preserved even at anesthetic concentrations that induce BS: In the primary somatosensory cortex of rats, EEG bursts were regularly accompanied by positive responses in the regional flow signal measured with a laser Doppler probe (Liu et al., 2011). Similarly, in anesthetized humans exhibiting BS EEG, transcranial Doppler ultrasound of the middle cerebral artery revealed that blood flow waxes throughout the burst phase and wanes during EEG suppression (Kuroda, Murakami, Tsuruta, Murakawa, & Sakabe, 1997). For many brain regions, our data reinforce that neurovascular coupling is preserved at sevoflurane‐induced BS. We observed global coherence between EEG and BOLD signal with a change of state in the EEG being followed by a change in the BOLD signal. The positive correlation of the two time courses in most of the gray matter complies with the commonplace understanding of neurovascular coupling (Kim & Ogawa, 2012; Lauritzen, Mathiesen, Schaefer, & Thomsen, 2012). However, the negative correlation between electrical activity and BOLD signal observed in occipital parts of the brain steps out of line and demands a further explanation.

A negative change next to a positive change of BOLD signal in adjacent brain regions has been attributed to reallocation of cortical blood (Harel, Lee, Nagaoka, Kim, & Kim, 2002; Shmuel et al., 2002). In these fMRI studies of visual stimulation, neural activity was assumed to be increased (although not measured) in both brain regions but associated with opposite BOLD changes. For our paradigm, we consider a vascular steal‐based explanation unlikely, since the opposed hemodynamic responses appeared in areas that belong to the carotid and the vertebrobasilar territory and are thus supplied largely independently.

The term “negative BOLD response” is often used to describe a BOLD decrease in response to a stimulus that in fact elicits a decrease in neural activity (e.g., Goense, Merkle, & Logothetis, 2012; Huber et al., 2014; Boorman et al., 2015). On the contrary, we observed an increase of neural activity in association with a decrease of BOLD signal. We used neither sensory stimulation nor a behavioral task, but assessed the relationship between EEG activity and BOLD signal in BS. While in the awake resting state, decreases in BOLD signal have often been associated with changes in EEG frequency content (Laufs et al., 2006; Magri et al., 2012; Scheeringa et al., 2011; Scholvinck et al., 2010), we found spectral properties in occipital and anterior leads to be similar for burst and suppression phases (Figure 1a,b). Thus, a peculiarity in frequency composition of occipital EEG cannot be a mechanism behind the observed anticorrelation.

Depending on the interaction between metabolism and hemodynamics, diminished BOLD signals can reflect high neuronal activity, with blood flow not adequately rising to match oxygen consumption. This might come into consideration for the initial dip of a BOLD signal (Chen‐Bee, Agoncillo, Xiong, & Frostig, 2007; Yacoub et al., 2001), but also sustained negative hippocampal BOLD signals during bicuculline‐induced seizures in rats (Schridde et al., 2008). Applied to burst phases coupled with BOLD decreases in our experiment, this would mean that the relative increase of CBF related to the relative increase of CMRO_2_ is lower in the occipital cortex as compared to the rest of the brain. Such a regional specificity of neurovascular coupling is expected to depend on the type of synaptic input, on the type of activated neurons, and on the balance between vasodilator and vasoconstrictor mediators released (Lauritzen et al., 2012). Supporting the assumption of a regionally unique coupling of flow and metabolism under anesthesia, sevoflurane‐induced decreases of CBF and CMRO_2_ are pronounced in the occipital lobe (Kaisti et al., 2003). However, we presently cannot clarify the mechanism behind the negative BOLD response to EEG bursts because measures of CMRO_2_ and CBF were not obtained.

An increase of BOLD signal in response to onset of EEG suppression is a further unexpected finding in the occipital lobe. During this exceptionally low level of electrical activity induced by a volatile anesthetic, vasoconstriction might not exceed a certain degree, resulting in excess oxygenated hemoglobin and, thus, an increase of BOLD signal. Again, this hypothesis cannot be verified due to lack of values for oxygen consumption and blood supply.

The thalamus could not be attributed to one of the two correlation patterns as per our GLM analysis. This conflicts with experimental observations of coherent BOLD fluctuations of thalamic nuclei and the neocortex in rats exhibiting isoflurane‐induced BS (Liu et al., 2011). In synchronous cortical and subcortical electrical recordings, however, thalamic neurons were found to be less synchronized with EEG BS patterns than cortical neurons. Moreover, thalamic neurons maintained their electrical activity during cortical silence, which led to the assumption that thalamocortical circuits are functionally disconnected in BS (Steriade, Amzica, & Contreras, 1994). Consistently, a ROI‐based analysis of our unsegmented BS data showed that thalamocortical connectivity is suppressed to a large extent (Ranft et al., 2016). A thalamic relay of visual and auditory stimuli, however, has been proven to remain functional even at isoflurane‐induced BS (Hudetz & Imas, 2007; Land, Engler, Kral, & Engel, 2012). Furthermore, we cannot rule out that thalamic activation/deactivation was subthreshold for fMRI.

## 5 CONCLUSION

With the present data from sevoflurane‐induced BS, we provide an example how a negative BOLD signal can originate from an actual increase of neuronal activity and vice versa, suggesting that the relationship of BOLD and underlying electrical signal can radically differ between brain regions. As has been conceded previously, the fMRI signal might potentially confuse excitation and inhibition (Logothetis, 2008). This needs to be taken into consideration when interpreting the BOLD signal during particular experimental conditions.

## CONFLICT OF INTERESTS

The authors declare no competing financial interests.
